# Pathophysiological Roles of Two Intracellular P-Type ATPases: The Cancer-Associated Na^+^,K^+^-ATPase α3 Isoform and the Parkinson’s Disease-Related ATP13A2

**DOI:** 10.3390/ijms27041800

**Published:** 2026-02-13

**Authors:** Takuto Fujii, Takahiro Shimizu, Hideki Sakai

**Affiliations:** Department of Pharmaceutical Physiology, Faculty of Pharmaceutical Sciences, University of Toyama, Toyama 930-0194, Japan; fujiitk@pha.u-toyama.ac.jp (T.F.); takshimi@pha.u-toyama.ac.jp (T.S.)

**Keywords:** P-type ATPase, Na^+^,K^+^-ATPase, H^+^,K^+^-ATPase, ATP13A2, cancer, anoikis, Parkinson’s disease, lysosome, metastasis

## Abstract

P-type ATPases constitute a diverse superfamily of ATP-driven transporters essential for ion homeostasis, membrane asymmetry, and organelle function. Among them, the P2-type Na^+^,K^+^-ATPase and the P5-type ATP13A2 have recently emerged as key regulators of cancer progression and neurodegeneration, respectively. In this review, we highlight new insights into the pathological roles of the Na^+^,K^+^-ATPase α3 isoform (α3NaK) in malignant cells and ATP13A2 in Parkinson’s disease (PD). Cancer tissues frequently overexpress α3NaK which is aberrantly localized to intracellular vesicles and undergoes adhesion-dependent intracellular trafficking. Upon cell detachment, α3NaK translocates to the plasma membrane to sustain survival signaling, thereby promoting anoikis resistance and facilitating the persistence of circulating tumor cells (CTCs). Cardiac glycosides selectively inhibit α3NaK at nanomolar concentrations, suppressing cancer cell proliferation through GLUT1 endocytosis, metabolic inhibition, and downregulation of THADA and LAT1, ultimately inducing anoikis in CTCs and reducing metastasis in vivo. Conversely, *ATP13A2* is genetically linked to early-onset parkinsonism and regulates lysosomal integrity, polyamine homeostasis, and neuronal resilience. Recent animal studies demonstrate that adult-onset ATP13A2 loss causes progressive nigrostriatal degeneration, while heterozygous deficiency produces distinct age-dependent cognitive and α-synuclein phenotypes. Beyond its established role in polyamine transport, emerging evidence suggests that ATP13A2 can function as an H^+^,K^+^-ATPase-like transporter, contributing to proton and cation handling within the endolysosomal system. Together, these findings underscore the broader physiological and pathological significance of intracellular P-type K^+^-ATPases and highlight α3NaK and ATP13A2 as promising therapeutic targets in cancer metastasis and PD.

## 1. Introduction

Adenosine triphosphate (ATP) is widely recognized as the cellular energy currency. However, the energy derived from its hydrolysis is utilized not only for driving metabolic reactions but also for a wide array of physiological processes, including membrane transport, ion homeostasis, and the maintenance of organelle function. Central to these processes are ATPases, a diverse group of enzymes that support the functional integration of cellular activities by transporting specific substrates (e.g., ions, lipids, and polyamines) across biological membranes [1,2].

For example, the sarco/endoplasmic reticulum Ca^2+^-ATPase (SERCA), localized to the sarcoplasmic and endoplasmic reticulum, plays a pivotal role in intracellular calcium storage, contributing to finely tuned regulation of excitation–contraction coupling and signal transduction [3,4,5]. The vacuolar H^+^-ATPase, located on lysosomal membranes, facilitates the acidification required for activating hydrolytic enzymes, thereby serving as a key component of the intracellular degradation system [6,7,8,9]. Moreover, the mitochondrial F_0_/F_1_-ATPase generates ATP via oxidative phosphorylation, functioning as the primary energy source for the cell [10,11,12].

On the plasma membrane, Na^+^,K^+^-ATPase maintains sodium and potassium gradients, thereby establishing the membrane potential, and functions as a primary active transporter that provides the driving force for secondary active transport processes [13,14,15]. The plasma membrane Ca^2+^-ATPase (PMCA) extrudes calcium from the cytosol to preserve intracellular calcium homeostasis and prevent calcium-induced cytotoxicity [16,17,18]. The H^+^,K^+^-ATPase expressed in gastric parietal cells mediates the final step of gastric acid secretion and supports digestive function [19,20,21,22,23].

**Figure 1:**
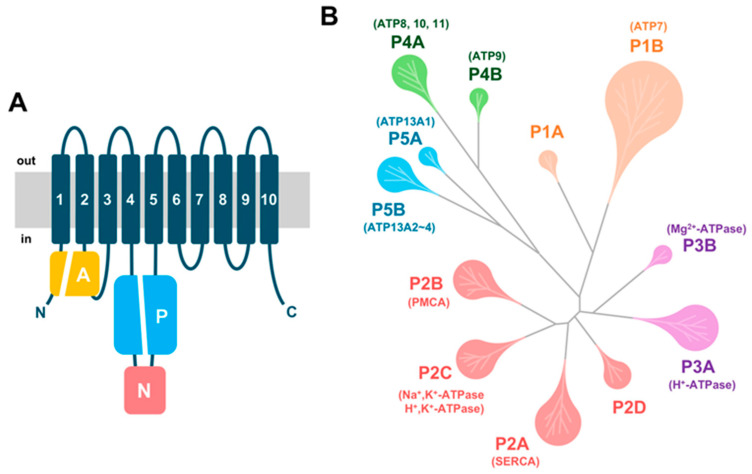
Overview of P-type ATPase domains and families. (**A**) Catalytic subunits of P-type ATPases typically contain ten transmembrane helices and three conserved cytoplasmic domains: the actuator (A) domain, the phosphorylation (P) domain containing the conserved phosphorylated aspartate, and the nucleotide-binding (N) domain, which harbors the ATP-binding site. The dark blue N and C at the ends of the topology represent the N-terminus and C-terminus, respectively. (**B**) Simplified phylogenetic tree of the P-type ATPase superfamily, with representative members indicated for each family. The Na^+^,K^+^-ATPase α3 isoform (α3NaK) and ATP13A2 belong to the P2C and P5B superfamilies, respectively.

Among the various classes of ATPases, P-type ATPases are characterized by ten transmembrane helices, and three cytoplasmic domains: the actuator (A), phosphorylation (P), and nucleotide-binding (N) domains (Figure 1A) [24]. These domains work in concert to couple ATP hydrolysis with the active transport of substrates across cellular membranes. A hallmark of P-type ATPases is the transient phosphorylation of a conserved aspartate residue during their catalytic cycle, a feature that distinguishes them from other ATPase families. Based on substrate specificity and sequence homology, P-type ATPases are classified into five major subgroups, P1 through P5, each fulfilling distinct physiological functions (Figure 1B) [25,26].

P1-type ATPases primarily transport copper ions, playing essential roles in metal homeostasis and detoxification. P2-type ATPases encompass well-characterized transporters such as Na^+^,K^+^-ATPase, H^+^,K^+^-ATPase, and Ca^2+^-ATPases (PMCA and SERCA) [27]. P3-type ATPases facilitate the transport of protons and magnesium ions, contributing to pH regulation and magnesium homeostasis [28,29,30]. P4-type ATPases, including ATP11A-C, are involved in the translocation of aminophospholipids, thereby regulating membrane asymmetry and vesicular trafficking [31,32]. P5-type ATPases, such as ATP13A1-5, remain less well characterized but are implicated in polyamine transport and organellar function [33].

This classification underscores the evolutionary diversification of P-type ATPases and their indispensable roles in cellular physiology. A comprehensive understanding of their molecular mechanisms and regulatory networks is crucial not only for elucidating fundamental biological processes but also for identifying novel therapeutic targets in diseases linked to ion transport dysfunction [34,35,36,37,38,39,40,41,42,43]. Recent advances have highlighted the diverse roles of intracellular P-type ATPases in disease pathogenesis beyond their canonical transport functions. In this review, we focus on two intracellular ATPases that transport potassium ions (K^+^), the P2-type Na^+^,K^+^-ATPase α3 isoform (α3NaK) in cancer cells and the P5-type ATP13A2 (PARK9) in neurons, to explore their emerging contributions to oncogenic and neurodegenerative processes.

## 2. Na^+^,K^+^-ATPase and Cardiac Glycosides

### 2.1. Na^+^,K^+^-ATPase

Na^+^,K^+^-ATPase is a transmembrane enzyme essential for maintaining the electrochemical gradient across the plasma membrane of animal cells [44,45]. It functions by actively transporting three sodium ions out of the cell and two potassium ions into the cell, a process driven by the hydrolysis of ATP to ADP. This electrogenic exchange not only sustains the resting membrane potential but also underpins a wide range of physiological processes, including neuronal excitability, muscle contraction, and epithelial transport [41,46,47]. Structurally, Na^+^,K^+^-ATPase is a heterodimer composed of a catalytic α-subunit and a glycosylated β-subunit, with an additional regulatory FXYD γ-subunit that modulates pump kinetics and tissue-specific activity [48,49]. The α-subunit contains binding sites for Na^+^,K^+^, ATP, and cardiac glycosides, and undergoes conformational cycling between the E1 and E2 states to mediate ion translocation; in comparison, the β-subunit is essential for proper folding, membrane trafficking, and stabilization of the α-subunit within the cell membrane.

A defining feature of Na^+^,K^+^-ATPase is the existence of four α isoforms (α1–α4; ATP1A1–ATP1A4), each exhibiting distinct tissue distribution, kinetic properties, and physiological roles [50,51]. The α1 isoform is expressed in nearly all cell types and functions as the housekeeping pump responsible for basal ion homeostasis. The α2 isoform, enriched in cardiac and skeletal muscle, in addition to adipose tissue, contributes to excitation–contraction coupling and metabolic regulation, and its altered activity has been implicated in cardiac arrhythmogenesis and hypertension [52,53,54]. The α3 isoform is predominantly expressed in neurons and is characterized by a lower affinity for Na^+^ and specialized for rapid restoration of ion gradients following high-frequency firing; mutations in *ATP1A3* are associated with severe neurological disorders, including alternating hemiplegia of childhood and rapid-onset dystonia–parkinsonism [55,56,57,58,59,60]. The α4 isoform, uniquely expressed in the testis, is essential for sperm motility and male fertility, reflecting a highly specialized adaptation of the pump to reproductive physiology [61,62,63].

Beyond its canonical ion-transporting function, Na^+^,K^+^-ATPase also serves as a receptor for cardiac glycosides, forming signaling complexes with Src family kinases and participating in pathways that regulate cell growth, apoptosis, and epithelial junction integrity [47,64,65]. Its signal-transducing activity is dynamically regulated by hormonal signals, intracellular Ca^2+^ signals, membrane lipid composition, and trafficking mechanisms that control pump abundance at the cell surface. Dysregulation of Na^+^,K^+^-ATPase has been implicated in diverse pathological conditions, including heart failure, ischemia–reperfusion injury, metabolic syndrome, neurodegenerative diseases, and cancer, underscoring its broad physiological significance [66].

### 2.2. Cardiac Glycosides

Cardiac glycosides represent an evolutionarily conserved and chemically diverse class of steroidal metabolites found in both plant and animal lineages, unified by their high-affinity inhibition of Na^+^,K^+^-ATPase and distinguished by the structure of their lactone ring [67,68]. These compounds share a conserved steroid nucleus glycosylated at C-3 but diverge into two major subclasses based on the lactone moiety: cardenolides, which possess a five-membered α,β-unsaturated butenolide ring, and bufadienolides, which are characterized by a six-membered doubly unsaturated α-pyrone ring [69]. Plant-derived cardiac glycosides are predominantly cardenolides and are widely distributed among species of Digitalis, Nerium, Thevetia, Calotropis, and Asclepias, where they function as specialized defensive secondary metabolites [70,71]. Their biosynthesis involves the modification of phytosterol precursors through a series of oxidation, glycosylation, and lactone-forming reactions, generating a remarkable structural diversity that influences lipophilicity, membrane permeability, and binding affinity to Na^+^,K^+^-ATPase.

Animal-derived cardiac glycosides, in contrast, are dominated by bufadienolides, which are synthesized in the parotoid glands and skin secretions of toads (*Bufo* and *Rhinella* spp.), where they serve as potent antipredator toxins [72,73,74]. Their biosynthetic origin remains less well understood than that of plant cardenolides; however, evidence suggests that they are derived from cholesterol-like precursors followed by lactone ring formation unique to amphibian metabolism. Beyond amphibians, bufadienolide-like molecules have been reported in certain insects and even in mammalian tissues, raising the possibility of endogenous biosynthesis or dietary acquisition [75,76,77,78]. Despite their distinct evolutionary origins, both cardenolides and bufadienolides share the same primary molecular target, and subtle structural differences in their lactone rings and glycosylation patterns contribute to variations in potency, isoform selectivity, and pharmacokinetics.

Pharmacologically, cardiac glycosides exert their classical positive inotropic effect in cardiomyocytes by inhibiting Na^+^,K^+^-ATPase, leading to intracellular Na^+^ accumulation and secondary elevation of Ca^2+^ via the Na^+^/Ca^2+^ exchanger [79]. This mechanism underlies the long-standing clinical use of digoxin and related cardenolides in the treatment of heart failure and certain arrhythmias. However, contemporary research has expanded the biological scope of cardiac glycosides far beyond their cardiotonic activity. These compounds modulate intracellular signaling pathways, including Src, MAPK, and NF-κB cascades; influence immune cell activation; and induce apoptosis or immunogenic cell death in cancer models [41,46,80,81,82]. Such pleiotropic actions have stimulated interest in their potential as anticancer, antiviral, and immunomodulatory agents, while also prompting efforts to engineer derivatives with improved therapeutic windows. Advances in synthetic biology, metabolic engineering, and semisynthetic modification have enabled access to rare or novel glycoside structures, facilitating systematic exploration of structure –activity relationships and the development of analogues with enhanced selectivity or reduced toxicity [68].

## 3. Na^+^,K^+^-ATPase α3 Isoform (α3NaK) in Cancer Cells

### 3.1. Intracellular α3NaK Movement Sustains Detached Cancer Cell Survival

Significant expression of α3NaK has been observed across various human cancer tissues, including those of the lung, pancreas, breast, bladder, bile duct, thyroid, ovary, colon, uterine body, stomach, and prostate. Notably, in one study, the proportion of α3NaK-positive tissues was found to be particularly high in colorectal (94%) and gastric (83%) cancers [83].

**Figure 2:**
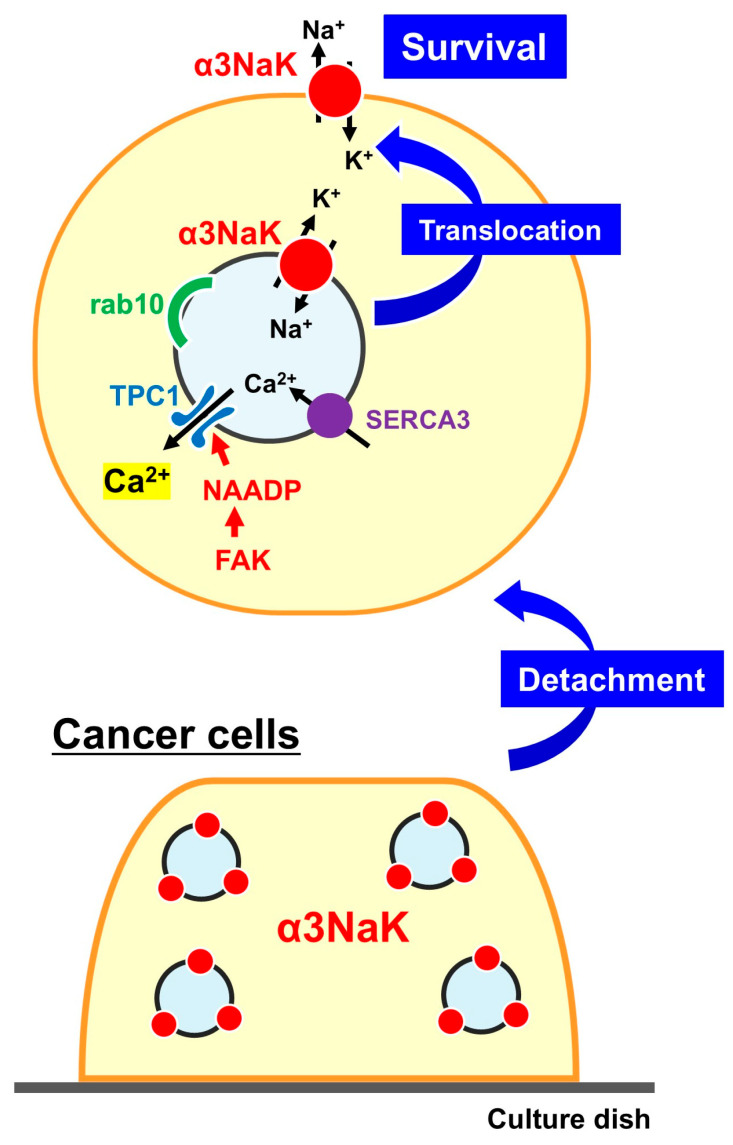
Cell detachment-induced translocation of α3NaK in cancer cells. In cells detached from the culture dish, α3NaK localized within intracellular vesicles containing rab10 and SERCA3 translocates to the plasma membrane through mechanisms involving FAK, NAADP, and Ca^2+^ channels. This mechanism is essential for cancer cell survival.

In immunohistochemical analysis of colorectal cancer and adjacent non-cancer tissues from patients, α3NaK was predominantly expressed in the cytoplasm of cancer cells, whereas little to no expression was detected in non-cancer cells. In HT-29 human colorectal cancer cells, the subcellular localization of α3NaK was found to be dynamically regulated by cell adhesion status (Figure 2). Under adherent conditions, α3NaK predominantly localized in the cytoplasm. Upon detachment from the culture dish using trypsin-EDTA, α3NaK translocated to the plasma membrane, as indicated by co-localization with the membrane marker flotillin-2. Remarkably, when detached cells were re-attached for 60 min, α3NaK returned to the cytoplasm. A similar trafficking pattern was observed in MKN45 human gastric cancer cells and HepG2 human liver cancer cells. These findings suggest that α3NaK trafficking is reversible and responsive to changes in cell adhesion.

Electron microscopy and schematic analysis results revealed that α3NaK is abnormally localized within intracellular vesicles situated directly beneath the plasma membrane in HT-29 cells. Importantly, the translocation of α3NaK-containing vesicles to the cell surface is orchestrated by vesicular trafficking and calcium-signaling components, including a small GTPase Rab10, SERCA3, nicotinic acid adenine dinucleotide phosphate (NAADP), and focal adhesion kinase (FAK). Following cell detachment, FAK-dependent and NAADP-mediated Ca^2+^ signaling promotes the fusion of α3NaK-containing vesicles—which are positive for Rab10 and SERCA3—with the plasma membrane, resulting in the translocation of α3NaK to the cell surface. Membrane translocation of α3NaK contributes to activation of AMP-activated protein kinase (AMPK) [83]. This regulated mobilization of α3NaK appears to constitute a critical adaptive mechanism that supports the survival of detached cancer cells during metastasis.

### 3.2. Anticancer Effect of Cardiac Glycosides

Epidemiological data suggest that cancer patients receiving cardiac glycosides, such as digoxin, exhibit improved overall survival compared with untreated patients [84]. This observation is supported by the results of retrospective analyses showing enhanced survival curves in digoxin-treated cohorts. Mechanistically, cardiac glycosides have been implicated in multiple anticancer pathways, including the include inhibition of cell proliferation [85,86], induction of apoptosis [87,88,89], enhancement of antitumor immune responses [84], and suppression of metastatic potential [90,91,92].

The results of recent studies have demonstrated that cardiac glycosides such as digoxin can selectively inhibit α3NaK at sub-micromolar concentrations, eliciting multiple anticancer effects [93,94,95]. In contrast, inhibition of α1NaK typically requires concentrations exceeding 2 μM [96], suggesting a therapeutic window in which cancer cells may be preferentially targeted while healthy tissues are relatively spared. Experimental data support this concept: digoxin treatment leads to dose-dependent suppression of cancer cell proliferation, with significant effects observed at concentrations as low as 30 nM [93]. These findings implicate α3NaK as a key mediator of the antitumor activity of cardiac glycosides, potentially through the modulation of intracellular signaling pathways unique to malignant cells.

### 3.3. Enzymatic Activity of α3NaK in Cancer Cells

The enzymatic activity of α3NaK in cancer cells was assessed by taking advantage of its lower affinity for Na^+^ compared with the ubiquitous α1NaK. Under low-Na^+^ conditions (20 mM), Na^+^,K^+^-ATPase activity is derived predominantly from α1NaK; in comparison, under high-Na^+^ conditions (120 mM), both α1NaK and α3NaK contribute to the total activity. Thus, α3NaK-specific activity can be estimated by subtracting the activity measured at 20 mM Na^+^ from that measured at 120 mM Na^+^ [97]. Using this approach, α3NaK-derived Na^+^,K^+^-ATPase activity was detected in the human gastric cancer cell line MKN45 and in human primary gastric cancer tissues [93]. Notably, this α3NaK-dependent activity was suppressed by 20 nM digoxin; in comparison, α1NaK-derived activity was unaffected at this concentration.

### 3.4. GLUT1 Endocytosis Induced by Cardiac Glycosides Through α3NaK

**Figure 3:**
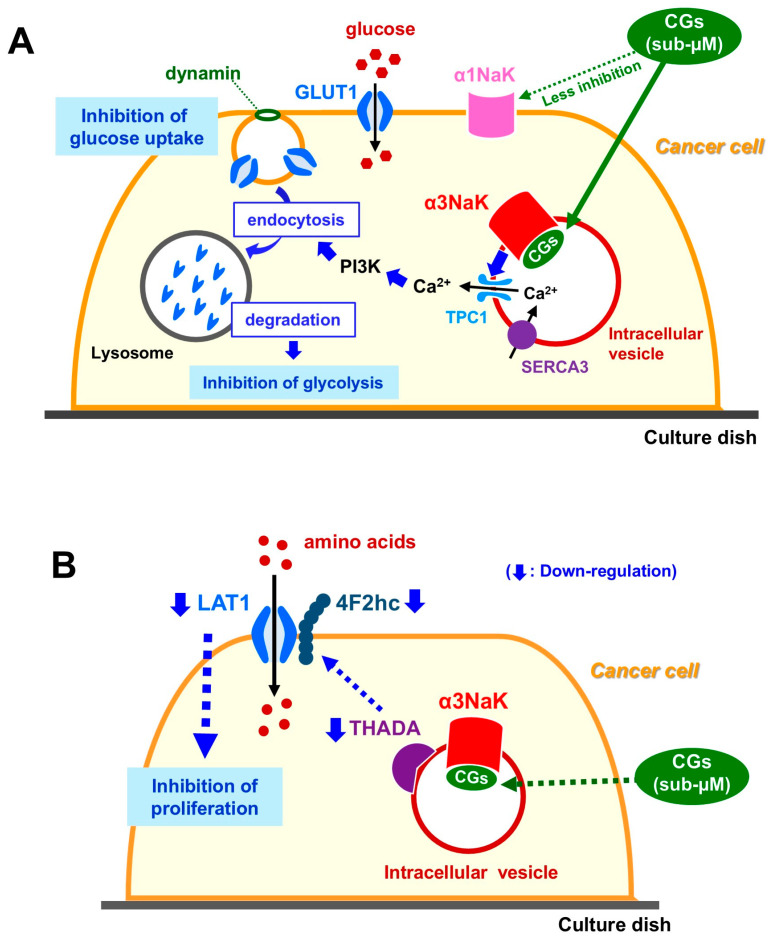
(**A**) Inhibition of glucose uptake and glycolysis by cardiac glycosides (CGs). CGs at sub-μM concentrations bind to α3NaK in intracellular vesicles containing TPC1 and SERCA3, rather than to α1NaK at the plasma membrane, thereby inducing dynamin-dependent endocytosis of the glucose transporter GLUT1. This response is initiated by Ca^2+^ release from the vesicles, which subsequently activates the PI3K pathway. The internalized GLUT1 is then trafficked to lysosomes for degradation, resulting in reduced glucose uptake. Collectively, these events suppress glycolysis in cancer cells. (**B**) Suppression of amino acid uptake and cell proliferation by CGs. At sub-μM concentrations, CGs interact with intracellular α3NaK, leading to downregulation of THADA and the amino acid transporter LAT1 and its associated protein, 4F2hc. This process amino acid uptake and intracellular nutrient availability, ultimately inhibiting cancer cell proliferation. The blue dashed arrows indicate the direction of the pathway after cardiac glycosides bind to α3NaK.

In recent studies, cardiac glycosides have been identified as unexpected modulators of cancer cell metabolism, acting through a previously unrecognized mechanism involving glucose transporter 1 (GLUT1) regulation [95]. Cardiac glycosides such as ouabain, oleandrin, and digoxin markedly reduce plasma membrane GLUT1 levels across multiple human cancer cell lines. Notably, ouabain decreased GLUT1 protein expression in a dose-dependent manner at concentrations ranging from 20 nM to 2 μM. This downregulation of GLUT1 was accompanied by a substantial decline in glucose uptake and extracellular lactate production, indicating impaired glycolytic flux (Figure 3A).

Mechanistically, these effects occurred at concentrations lower than those required to inhibit the canonical α1NaK activity. Instead, the aberrantly expressed intracellular α3NaK was identified as the critical target. Binding of cardiac glycosides to α3NaK triggered NAADP-dependent Ca^2+^ release via two-pore channel 1 (TPC1), which in turn activated phosphoinositide 3-kinase (PI3K). This signaling cascade promoted dynamin-dependent endocytosis and lysosomal degradation of GLUT1, leading to marked inhibition of glucose uptake, glycolytic activity, and cell proliferation. Collectively, these findings reveal a novel α3NaK-mediated pathway by which cardiac glycosides suppress aerobic glycolysis in cancer cells, highlighting GLUT1 and its regulatory axis as promising therapeutic targets in malignancies characterized by elevated glycolytic demand.

### 3.5. THADA Downregulation in Cardiac Glycoside Anticancer Effects

Concerning the downstream effectors that mediate the anti-proliferative actions of cardiac glycosides, recent studies have provided important mechanistic insight with the identification of thyroid adenoma-associated protein (THADA) as a key molecule suppressed by cardiac glycosides and required for cancer cell growth [94]. THADA, originally discovered as a gene disrupted in thyroid adenomas, has been implicated in diverse biological processes including thermogenesis, energy homeostasis, and insulin secretion [98,99,100]. Its functional association with P-type ATPases such as SERCA suggests that THADA may act as a regulator of intracellular ion handling and metabolic balance. More recently, THADA has been linked to cancer biology, including associations with prostate cancer risk and promotion of programmed cell death ligand 1 (PD-L1) maturation, indicating a broader role in tumor progression and immune regulation [101].

Cardiac glycosides such as ouabain, oleandrin, and digoxin markedly reduce THADA expression at both the mRNA and protein levels in human cancer cells [94]. THADA knockdown suppresses cancer cell growth and its re-expression restores proliferative capacity. Through transcriptomic analysis, the L-type amino acid transporter LAT1 (SLC7A5) and its chaperone 4F2hc (SLC3A2) were identified as downstream targets of THADA. As LAT1 is a major transporter of essential amino acids and is widely upregulated in aggressive cancers, its downregulation provides a plausible metabolic mechanism for the anti-proliferative effects observed. In line with these findings, cardiac glycosides also suppress LAT1 and 4F2hc expression, and pharmacological inhibition of LAT1 reduces cancer cell growth (Figure 3B).

A notable finding is that THADA colocalizes with the intracellular α3NaK rather than the plasma membrane α1NaK. Inhibition of intracellular α3NaK triggers downregulation of THADA, which in turn reduces LAT1 expression and amino acid uptake. This proposed α3NaK-THADA-LAT1 axis provides a coherent mechanistic framework linking Na^+^,K^+^-ATPase inhibition to metabolic suppression in cancer cells.

## 4. Anoikis Resistance Mediated by α3NaK and Its Role in Cancer Metastasis

### 4.1. Anoikis

Metastasis, the spread of cancer cells from the primary tumor to distant organs, depends on the ability of cells to survive detachment from the extracellular matrix (ECM). This detachment typically induces a specialized form of programmed cell death known as anoikis [102,103]. In healthy epithelial tissues, loss of ECM attachment triggers anoikis, thereby preventing inappropriate cell migration and colonization. In contrast, cancer cells often develop resistance to anoikis, allowing them to remain viable in suspension, enter blood or lymphatic vessels, and eventually form secondary tumors in remote tissues [104,105].

Anoikis resistance arises from a complex network of signaling alterations, including changes in integrin-mediated adhesion, activation of survival pathways such as PI3K/Akt and MAPK, and regulation of apoptotic machinery [106,107,108]. These adaptations enable cancer cells to evade the death signals that would normally follow matrix detachment. Although the phenotypic outcomes of anoikis resistance are well characterized, its full molecular basis remains to be elucidated. Clarifying the mechanisms underlying anoikis resistance is critical for understanding the metastatic process and identifying therapeutic strategies to inhibit cancer dissemination.

### 4.2. Circulating Tumor Cells (CTCs)

CTCs are malignant cells that detach from the primary tumor mass and enter the peripheral bloodstream [109,110,111]. This process, often referred to as tumor cell intravasation, represents a critical step in the metastatic cascade. Once in circulation, CTCs possess the potential to extravasate into distant tissues, where they may initiate the formation of secondary tumors. As such, CTCs are considered a major cellular source of cancer recurrence and metastasis, and their presence reflects the dynamic and invasive nature of malignant disease.

Accumulating clinical evidence has underscored the prognostic relevance of CTC enumeration. In particular, the results of a landmark study by Cristofanilli et al. [112] demonstrated that patients with metastatic breast cancer harboring ≥5 CTCs per 7.5 mL of blood exhibited significantly reduced overall survival compared to those with fewer than 5 CTCs. This survival disparity, visualized through Kaplan–Meier analysis, highlights the utility of CTC counts as a quantitative biomarker for disease aggressiveness and therapeutic response. These results raise the possibility that the α3NaK trafficking described above contributes to anoikis resistance in CTCs, as discussed in the following section.

Beyond their prognostic value, CTCs have emerged as a cornerstone of liquid biopsy, a minimally invasive approach that enables real-time monitoring of tumor biology [113,114,115]. Analysis of CTCs enables early detection of disease progression, prediction of metastatic potential, and evaluation of treatment efficacy. Importantly, CTCs can be isolated and characterized at the molecular level, providing insights into tumor heterogeneity, mechanisms of drug resistance, and actionable genetic alterations. These attributes position CTCs as a powerful tool in the era of precision oncology.

### 4.3. CTC Anoikis Induced by Digoxin via α3NaK

**Figure 4:**
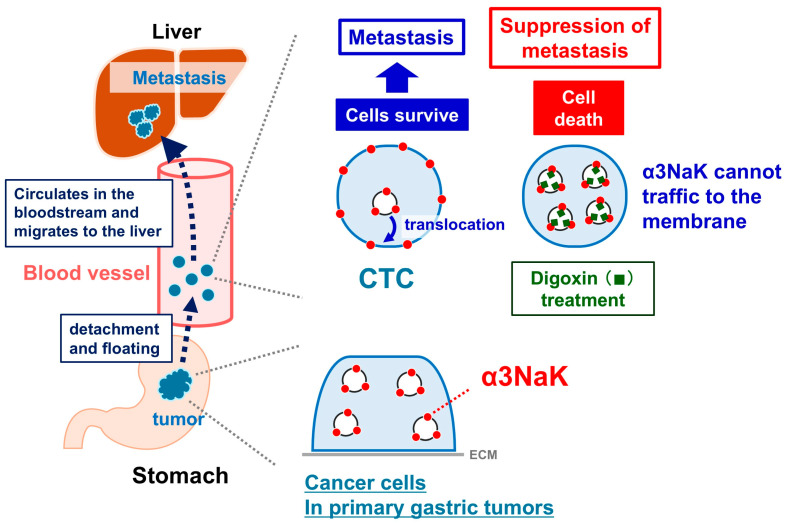
Digoxin treatment suppresses circulating tumor cell (CTC)-mediated metastasis. Schematic illustration of the metastatic progression in gastric cancer, highlighting the role of CTCs. Cancer cells detach from the primary gastric tumors and enter the bloodstream as CTCs, which translocate α3NaK to the plasma membrane to promote survival and liver metastasis. Digoxin treatment inhibits α3NaK membrane trafficking, resulting in CTC death and suppression of metastasis.

In CTCs isolated from gastric cancer patients and in mouse xenograft models, α3NaK is predominantly localized at the plasma membrane under detached conditions; in comparison, it relocates to the cytoplasm upon reattachment [93]. This detachment-induced translocation appears to be essential for CTC survival, as CTCs express integrin α5 [116,117,118] but lack fibronectin [119,120], suggesting that loss of ECM engagement triggers α3NaK movement to the plasma membrane as a critical mechanism for anoikis resistance (Figure 4).

In human MKN45 gastric cancer cells, digoxin at nanomolar concentrations selectively inhibits α3NaK activity and blocks its translocation to the plasma membrane without affecting the ubiquitous α1NaK. This inhibition enhances caspase-3 and -7 activation and reduces viability specifically in detached cells, indicating that digoxin promotes anoikis by preventing α3NaK-dependent survival signaling. In vivo, digoxin administration (2 mg/kg/day in mice) markedly reduces the number of gastric CTCs in the bloodstream and suppresses liver metastasis in orthotopic and subcutaneous xenograft models, despite having little effect on primary tumor size [93].

Collectively, these findings indicate that α3NaK plays a pivotal role in the early stages of metastatic cascade by enabling CTC survival following loss of ECM attachment, and that pharmacological inhibition of α3NaK translocation represents a promising strategy to eliminate CTCs and prevent metastasis. The work also positions digoxin as a dual-acting agent in metastasis biology, both disrupting CTC clusters as reported previously [121] and inducing anoikis in single CTCs, highlighting its potential therapeutic relevance in targeting metastatic dissemination.

## 5. Parkinson’s Disease and Its Related Genes

### 5.1. Parkinson’s Disease

Parkinson’s disease (PD) is clinically defined by the progressive loss of dopaminergic neurons in the substantia nigra pars compacta, leading to striatal dopamine deficiency and the emergence of characteristic motor symptoms such as bradykinesia, rigidity, resting tremor, and postural instability [122,123]. Beyond these hallmark motor features, PD encompasses a wide array of nonmotor manifestations, including autonomic dysfunction, sleep disturbances, cognitive decline, and neuropsychiatric symptoms, which often appear before the onset of motor symptoms and significantly affect quality of life. Pathologically, the accumulation of misfolded α-synuclein within Lewy bodies and Lewy neurites represents a central hallmark, suggesting that protein aggregation and impaired cellular homeostasis play important roles in disease progression [124,125]. Epidemiologically, PD is one of the most common neurodegenerative disorders, with incidence increasing sharply with age, and it is thought to arise from a complex interplay of age-related vulnerabilities, environmental exposures, intrinsic cellular stress responses, and genetic predisposition, as demonstrated by the identification of multiple PD-associated genes and PARK loci [126,127].

### 5.2. PD-Related Genes

Over the past two decades, through extensive genetic studies, researchers have identified multiple loci associated with familial and sporadic forms of PD, shedding light on its complex etiology. To date, more than a dozen PARK loci have been mapped, each corresponding to distinct genes and protein products implicated in various pathogenic mechanisms, including α-synuclein (SNCA, PARK1/4), Parkin (PARK2), PINK1 (PARK6), DJ-1 (PARK7), and LRRK2 (PARK8), among others, with inheritance patterns ranging from autosomal dominant to autosomal recessive, and ages of onset spanning from juvenile or early-onset to late adulthood [128,129]. Notably, *ATP13A2* (*PARK9*), located on chromosome 1p36, has emerged as a causative gene for Kufor–Rakeb syndrome, a rare autosomal recessive form of early-onset parkinsonism [130,131]. *ATP13A2* encodes a lysosomal P-type ATPase with ten transmembrane domains, and pathogenic mutations have been linked to impaired lysosomal function and progressive neuronal degeneration [132].

## 6. Pathophysiological Characterization of ATP13A2 in Animal Models

### 6.1. Mouse Models

Erb et al. [133] provided a decisive clarification of the pathogenic consequences of ATP13A2 loss in the adult brain of mice. Although germline *Atp13a2* knockout (KO) mice exhibit lysosomal abnormalities and mild motor phenotypes without dopaminergic neurodegeneration, the authors demonstrate that the developmental timing of ATP13A2 depletion is critical. Using unilateral AAV-Cre delivery to the substantia nigra of conditional floxed mice, they achieved efficient adult-onset ATP13A2 deletion and observed progressive degeneration of the nigrostriatal dopaminergic pathway, beginning with early terminal loss at three months and culminating in substantial neuronal loss by ten months. This degeneration was accompanied by lysosomal enlargement, accumulation of LAMP2-positive vesicles, p62-positive inclusions, and transient neuroinflammatory responses, while α-synuclein and tau aggregation remained absent. These findings indicate that compensatory mechanisms likely protect germline knockouts during development. The authors propose that such compensatory pathways may involve the mitochondrial prosurvival factor Mcl-1, in addition to genes responsive to lysosomal stress or damage, and they emphasize that elucidating these mechanisms will be important for future studies.

Croucher et al. [134] conducted one of the most comprehensive in vivo examinations to date of how partial versus complete loss of ATP13A2 function shapes the trajectory of age-related neurobiological changes in mice. Although ATP13A2 has long been implicated in the pathogenesis of PD and related synucleinopathies, the field has lacked systematic comparisons between heterozygous (Het) and KO states, representing an important gap because many human carriers harbor heterozygous variants. The authors confirmed a graded reduction of *Atp13a2* mRNA across genotypes and reproduced the well-established KO phenotype, including age-dependent sensorimotor impairments, early reductions in striatal dopamine and serotonin, and robust, widespread gliosis. Notably, KO mice also exhibited pronounced alterations in polyamine homeostasis across multiple brain regions, consistent with ATP13A2’s role in lysosomal polyamine export. In contrast, Het mice displayed a distinct and previously underappreciated phenotype: early cognitive deficits in object recognition, followed by progressive and region-specific accumulation of α-synuclein and phosphorylated α-synuclein with aging. Strikingly, α-synuclein pathology was more prominent in Het than in KO mice across several brain regions, suggesting that partial ATP13A2 loss may exacerbate α-synuclein dysregulation more strongly than complete loss. This may be due to compensatory mechanisms in KO mice that are absent in Het mice. These findings reveal that ATP13A2 dosage exerts divergent effects on lysosomal function, polyamine metabolism, glial activation, and α-synuclein homeostasis. The study authors highlight that heterozygous states cannot be assumed to represent a simple intermediate between wild-type (WT) and KO, but instead may engage distinct pathogenic pathways.

In contrast to the above studies involving mouse models, Massari et al. [135] employed a well-established paradigm in which α-synuclein preformed fibrils are injected into the striatum of mice. They found that the distribution of pathological α-synuclein, nigrostriatal dopaminergic neurodegeneration, reactive astrogliosis, microglial activation, and lysosomal alterations were largely unchanged in KO mice compared with WT controls. These findings indicate that ATP13A2 loss-of-function does not exacerbate α-synuclein-driven pathology, suggesting that, at least in this model, ATP13A2-related lysosomal dysfunction may contribute to disease through mechanisms partly independent of α-synuclein aggregation.

### 6.2. Rat Model

Through the use of a CRISPR/Cas9-engineered *Atp13a2* KO rat, the first comprehensive phenotypic characterization was conducted, providing new insights into ATP13A2-linked pathophysiology [136]. The KO rats exhibited mild but significant neurodevelopmental delays, including postponed eye opening, delayed acoustic startle, and transient impairments in early locomotor milestones. In adulthood, these animals developed age-dependent motor abnormalities, including hyperactivity and deficits in fine motor coordination, while maintaining preserved nigrostriatal dopaminergic neuron numbers. Neurochemical analysis results revealed region- and age-specific alterations in glutamate, GABA, and dopamine metabolism, suggesting compensatory adaptations rather than overt neurodegeneration. This new rat model recapitulates key biochemical, lysosomal, and behavioral features of ATP13A2-related disease without spontaneous dopaminergic neuron loss and highlights the central role of ATP13A2 in autophagy-lysosomal pathway integrity, polyamine handling, and neuronal homeostasis.

### 6.3. Monkey Model

In a pilot study using non-human primates (rhesus monkey; *Macaca mulatta*), compelling evidence has been provided that ATP13A2 plays an essential role in maintaining dopaminergic neuron integrity [137]. Using bilateral lentiviral delivery of an ATP13A2 targeting shRNA into the substantia nigra of macaques, the study results suggest that partial depletion of ATP13A2 (~60%) is sufficient to trigger progressive nigrostriatal degeneration, including loss of the tyrosine hydroxylase-positive neurons in the substantia nigra and reduced dopaminergic markers in the caudate and putamen. In addition, ATP13A2 deficiency induces key pathological features associated with Parkinson’s disease and Kufor–Rakeb syndrome, such as increased phosphorylation of α-synuclein, accumulation of LAMP2-positive lysosomal and autophagic vesicles, lipofuscin deposition, and marked dysregulation of metal homeostasis, including nigral iron levels. These findings highlight the central role of ATP13A2 in lysosomal function, α-synuclein handling, and metal homeostasis.

## 7. Molecular and Physiological Functions of ATP13A2

### 7.1. Structural Basis of Polyamine Transport by ATP13A2

Polyamine transport by ATP13A2 was first demonstrated by Vangheluwe and colleagues, establishing ATP13A2 as a lysosomal polyamine exporter [138]. Building on this functional insight, in a cryo-EM study by Mu et al. [139], the authors provide a near-complete structural framework for the conformational cycle of human ATP13A2. By determining seven high-resolution structures representing six intermediate states, including the previously elusive E2 state, the sequential steps of substrate recruitment, translocation, and release are delineated. The study authors identify two distinct polyamine-binding sites and a large inward-facing cavity that likely functions as a substrate-buffering chamber, a feature that distinguishes ATP13A2 from other P-type ATPases.

Integration of structural analysis, biochemical assays, and multiscale molecular dynamics simulations reveals how ATP13A2 coordinates ATP-driven conformational changes with polyamine movement across the membrane [140,141,142,143]. The results of these studies also highlight the potential regulatory role of negatively charged phospholipids, such as PI(3,5)P_2_, which cluster near the release site and may facilitate substrate exit.

Together, these findings establish the most comprehensive mechanistic model to date for ATP13A2-mediated polyamine transport. This structural framework not only advances our understanding of lysosomal polyamine biology but also provides critical insights into how ATP13A2 dysfunction contributes to neurodegenerative disease.

### 7.2. Lysosomal H^+^,K^+^-ATPase Function of ATP13A2

**Figure 5:**
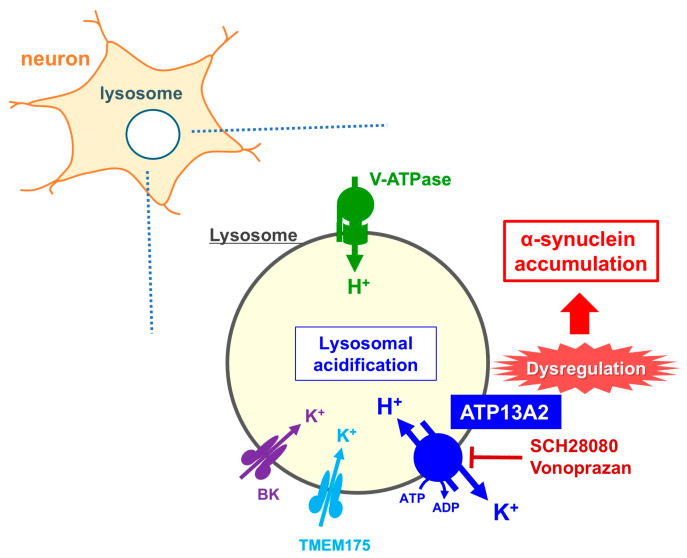
Contribution of ATP13A2 to lysosomal acidification and its dysregulation leading to α-synuclein accumulation in neurons. Schematic representation of lysosomal acidification mechanisms within a neuron. V-ATPase actively pumps H^+^ into the lysosomal lumen; in parallel, ATP13A2 also contributes to maintaining luminal acidity through its H^+^/K^+^ transport activity. K^+^ influx into the lysosome mediated by large-conductance calcium-activated potassium (BK) and TMEM175 channels may be functionally coupled to K^+^ efflux mediated by ATP13A2. Pharmacological inhibition of ATP13A2 by SCH28080 or vonoprazan disrupts lysosomal acidification, leading to lysosomal dysfunction and subsequent α-synuclein accumulation.

Although ATP13A2 has been implicated in lysosomal integrity, polyamine export, and protection against α-synuclein toxicity, its fundamental cation-transporting mechanism remained poorly understood until our recent study. We provide compelling biochemical and cellular evidence that ATP13A2 functions as a lysosomal H^+^,K^+^-ATPase, thereby establishing a previously unrecognized mechanism for lysosomal acid-base regulation [144] (Figure 5).

Using heterologous expression systems and *ATP13A2*-knockout cells, we demonstrate that ATP13A2 exhibits K^+^-dependent ATPase activity and mediates ATP-driven K^+^ efflux from the lysosomal lumen. Interestingly, this activity is selectively inhibited by a SERCA inhibitor, thapsigargin, and by potassium-competitive acid blockers (P-CABs) such as SCH28080 and vonoprazan, classic inhibitors of gastric H^+^,K^+^-ATPase (ATP4A). These inhibitors also induce lysosomal alkalinization and promote α-synuclein accumulation, two pathological features strongly associated with PD. However, no data are currently available regarding the blood–brain barrier permeability of P-CABs in humans. It has been reported that P-CABs do not meaningfully increase neurological adverse events such as headache or dizziness [145]. Thus, at present, P-CABs are unlikely to exert harmful effects in PD patients. In contrast, another type of gastric H^+^,K^+^-ATPase (ATP4A) inhibitor, omeprazole, exhibited no effect on ATP13A2 activity.

Several PD-linked ATP13A2 mutations have been reported to compromise lysosomal polyamine binding and transport [138,143]. Importantly, among these variants, we found that R449Q and A746T markedly reduce both ATPase activity and K^+^ transport, supporting the pathogenic relevance of impaired H^+^/K^+^ exchange. Because the function of ATP13A2 is also suppressed by the V-ATPase inhibitor bafilomycin [144], ATP13A2 and V-ATPase may be functionally interconnected. This finding suggests that V-ATPase and ATP13A2 act in a complementary manner, and that loss of ATP13A2 function likely has a substantial impact on the maintenance of acidification. However, further studies and experimental validation are required to clarify this regulatory relationship.

Our study results further demonstrate that ATP13A2 activity is enhanced under acidic luminal pH and is stimulated by spermidine, suggesting a functional coupling between polyamine export and H^+^/K^+^ transport. Together with the above-mentioned cryo-EM structures, these findings position ATP13A2 as a unique lysosomal pump that coordinates proton influx and potassium efflux to maintain luminal acidity, support autophagic degradation, and prevent α-synuclein aggregation.

Overall, this work resolves a long-standing question regarding the ion-transport mechanism of ATP13A2 and provides a mechanistic framework linking ATP13A2 dysfunction to lysosomal failure and PD pathogenesis. The identification of ATP13A2 as a lysosomal H^+^,K^+^-ATPase also raises important considerations for the neurological safety of gastric P-CABs and highlights lysosomal cation homeostasis as a potential therapeutic target in PD.

### 7.3. Glial Extracellular Vesicle Modulation by ATP13A2 in α-Synuclein Spread

Using *Atp13a2*-null mice and lentiviral overexpression models, Tsunemi et al. [146] demonstrated that ATP13A2 levels critically influence extracellular vesicle (EV) biogenesis in vivo. Loss of ATP13A2 reduced EV secretion but did not significantly alter α-synuclein preformed fibril (PFF) propagation in mouse brains. In contrast, ATP13A2 overexpression markedly increased EV release and unexpectedly attenuated both local and distant accumulation of α-synuclein pathology. This reduction was observed for total α-synuclein inclusions, in addition to phosphorylated α-synuclein, the pathological form associated with Lewy bodies and Lewy neurites. These findings suggest that enhancing endogenous EV secretion may facilitate the uptake and degradation of secreted α-synuclein PFFs by regional glial cells, thereby limiting their widespread propagation throughout the brain.

The results of time-course analyses revealed that inoculated α-synuclein PFFs were initially and preferentially taken up by microglia and astrocytes rather than neurons. Glial cells rapidly internalized PFFs and subsequently released them within EVs; in comparison, neurons exhibited slower uptake but ultimately accumulated phosphorylated α-synuclein. The results of in vitro experiments using iPSC-derived neurons and astrocytes together with primary microglia further demonstrated that glial cells secreted substantially more EVs than neurons and that glia-derived EVs were more efficiently internalized by neurons. ATP13A2 deficiency in any of these cell types decreased EV secretion and increased the α-synuclein fibril load per vesicle, suggesting impaired vesicular handling.

Collectively, these findings highlight a dual role for glial cells in α-synuclein propagation: they act as early phagocytic recipients of extracellular α-synuclein fibrils and subsequently as major sources of EV-mediated transfer to neurons. Importantly, enhancing ATP13A2-dependent EV secretion appears to promote the clearance of α-synuclein within glia and reduce its accumulation in neurons, thereby mitigating the spread of pathology. This work positions ATP13A2 and EV biogenesis as potential therapeutic targets for synucleinopathies.

### 7.4. Polyamine-Driven Impairment of Lysosomal β-Glucocerebrosidase in ATP13A2 Deficiency

Using *ATP13A2*-deficient cell lines, iPSC-derived neurons, and *Atp13a2* knockout mice, Samaddar et al. [147] demonstrate that loss of ATP13A2 leads to selective accumulation of polyamines within lysosomes, without altering their cytosolic levels. This primary lysosomal polyamine storage triggers secondary accumulation of bis(monoacylglycero)phosphate (BMP) and β-glucocerebrosidase (GCase) substrates, including glucosylsphingosine, in an age-dependent manner.

On a mechanistic basis, the study results reveal a dual inhibitory effect of excess lysosomal polyamines on GCase activity. First, the polybasic nature of polyamines causes lysosomal deacidification, reducing the enzyme’s catalytic efficiency. Second, their polycationic charge interferes with electrostatic interactions between GCase and the anionic lipid BMP, which are essential for interfacial catalysis on intraluminal vesicles. These mechanisms were validated using a liposome-based cell-free assay, wherein polyamines dose-dependently inhibited BMP-stimulated GCase activity, and BMP supplementation partially rescued this inhibition.

Together, these findings highlight lysosomal polyamine overload as a newly identified factor driving glycosphingolipid storage and link ATP13A2 dysfunction to impaired GCase activity. The above findings suggest that modulating lysosomal pH or BMP levels may offer therapeutic avenues for ATP13A2-associated neurodegeneration.

## 8. Conclusions and Future Directions

P-type ATPases constitute a highly conserved and functionally diverse family of membrane transporters that are indispensable for maintaining cellular ion gradients, membrane asymmetry, and organellar homeostasis. Recent advances have expanded our understanding of these ATPases beyond their canonical transport functions, revealing unexpected roles in intracellular signaling, metabolic regulation, and disease pathogenesis. In this review, we highlight two intracellular P-type ATPases, the Na^+^,K^+^-ATPase α3 isoform (α3NaK) and ATP13A2, as emerging regulators of cancer progression and metastasis, and neurodegeneration, respectively.

Accumulating evidence indicates that α3NaK is aberrantly expressed in a wide range of human cancers and undergoes dynamic intracellular trafficking that enables detached cancer cells, including CTCs, to evade anoikis. Cardiac glycosides selectively targeting α3NaK at nanomolar concentrations suppress cancer cell proliferation, induce GLUT1 endocytosis, disrupt amino acid uptake through THADA-LAT1 downregulation, and promote anoikis in CTCs. These findings position α3NaK as a promising therapeutic vulnerability in metastatic disease and suggest that repurposing cardiac glycosides or developing α3NaK-selective inhibitors may offer new strategies to eliminate disseminating cancer cells.

In parallel, investigation into ATP13A2 have deepened our understanding of lysosomal dysfunction in Parkinson’s disease. Conditional and heterozygous mouse models, in addition to CRISPR-engineered rats, demonstrate that ATP13A2 loss impairs lysosomal integrity, disrupts polyamine homeostasis, and contributes to age-dependent neurodegeneration. Importantly, recent biochemical insights suggest that ATP13A2 may function as a H^+^,K^+^-ATPase-like transporter in lysosomes, mediating proton–cation exchange and contributing to luminal pH regulation. This emerging concept provides a mechanistic framework linking ATP13A2 deficiency to lysosomal alkalinization, impaired proteostasis, and selective dopaminergic vulnerability. Notably, partial ATP13A2 deficiency can provoke distinct pathological outcomes compared with complete loss, underscoring the importance of ATP13A2 dosage in neuronal resilience.

Despite these advances, several key questions remain unresolved. Regarding α3NaK, the molecular determinants such as transcription factors and epigenetic modifications that regulate its cancer-specific expression, intracellular trafficking machinery, and signaling outputs require further elucidation. Understanding how α3NaK integrates with metabolic and immune pathways may reveal additional therapeutic opportunities. Regarding ATP13A2, defining its precise transport mechanism, including the extent to which its H^+^,K^+^-ATPase-like activity contributes to lysosomal acid-base homeostasis, remains a central challenge. Moreover, the divergent phenotypes observed in heterozygous versus knockout models call for deeper investigation into compensatory pathways and gene-environment interactions.

Future studies integrating structural biology, single-cell omics, advanced imaging, and in vivo disease modeling will be crucial for unraveling the multifaceted roles of P-type ATPases in human health and disease. Ultimately, translating these mechanistic insights into targeted therapeutics may open new avenues for combating metastatic cancer and neurodegenerative disorders.

## Data Availability

No new data were created or analyzed in this study. Data sharing is not applicable to this article.

## Abbreviations

The following abbreviations are used in this manuscript:

α3NaK	Na^+^,K^+^-ATPase α3 isoform
AMPK	AMP-activated protein kinase
ATP	Adenosine triphosphate
BMP	bis(monoacylglycero)phosphate
CTCs	circulating tumor cells
ECM	extracellular matrix
EV	extracellular vesicle
FAK	focal adhesion kinase
GCase	β-glucocerebrosidase
GLUT1	Glucose transporter 1
Het	heterozygous
KO	knockout
LAT1	L-type amino acid transporter 1
NAADP	nicotinic acid adenine dinucleotide phosphate
P-CABs	potassium-competitive acid blockers
PD	Parkinson’s disease
PFF	preformed fibril
PI3K	phosphoinositide 3-kinase
PMCA	plasma membrane Ca^2+^-ATPase
SERCA	sarco/endoplasmic reticulum Ca^2+^-ATPase
THADA	thyroid adenoma-associated protein
TPC1	two-pore channel 1
WT	wild-type
